# Moderately hypofractionated radiotherapy as definitive treatment for localized prostate cancer: Pattern of practice in German-speaking countries: A survey of the Prostate Cancer Expert Panel of the German Society of Radiation Oncology (DEGRO) and the Working Party on Radiation Oncology of the German Cancer Society (DKG-ARO)

**DOI:** 10.1007/s00066-021-01820-2

**Published:** 2021-08-31

**Authors:** Mohamed Shelan, Daniel M. Aebersold, Clemens Albrecht, Dirk Böhmer, Michael Flentje, Ute Ganswindt, Stefan Höcht, Tobias Hölscher, Arndt-Christian Müller, Peter Niehoff, Michael Pinkawa, Nina-Sophie Schmidt-Hegemann, Felix Sedlmayer, Frank Wolf, Constantinos Zamboglou, Daniel Zips, Thomas Wiegel, Pirus Ghadjar

**Affiliations:** 1 Universitätsklinik für Radio-Onkologie, Inselspital, Universität Bern, Bern, Switzerland; 2 Klinik für Radioonkologie und Gemeinschaftspraxis für Strahlentherapie, Klinikum Nürnberg Nord, Universitätsklinikum der Paracelsus Medizinischen Privatuniversität, Nuremberg, Germany; 3 Klinik für Radioonkologie und Strahlentherapie, Charité Universitätsmedizin Berlin, corporate member of Freie Universität Berlin, Humboldt-Universität zu Berlin, and Berlin Institute of Health, Augustenburger Platz 1, 13353 Berlin, Germany; 4 Klinik und Poliklinik für Strahlentherapie, Universitätsklinikum Würzburg, Würzburg, Germany; 5 Universitätsklinik für Strahlentherapie-Radioonkologie, Innsbruck, Austria; 6 Xcare Praxis für Strahlentherapie Saarlouis, Xcare Gruppe, Saarlouis, Germany; 7 Klinik und Poliklinik für Strahlentherapie und Radioonkologie, Universitätsklinikum Carl Gustav Carus, Technische Universität Dresden, Dresden, Germany; 8 Universitätsklinik für Radioonkologie, Universitätsklinikum Tübingen, Tübingen, Germany; 9 Sana Klinikum Offenbach, Offenbach, Germany; 10 MediClin Robert Janker Klinik, Bonn, Germany; 11 Klinik und Poliklinik für Strahlentherapie und Radioonkologie, LMU Klinikum, Munich, Germany; 12 Universitätsklinik für Radiotherapie und Radio-Onkologie, LKH, Universitätsklinikum der Paracelsus Medizinischen Privatuniversität, Müllner Hauptstraße 48, 5020 Salzburg, Austria; 13 Klinik für Radio-Onkologie, Universitätsklinikum Freiburg, Freiburg, Germany; 14 Klinik für Radioonkologie und Strahlentherapie, Universitätsklinikum Ulm, Ulm, Germany

**Keywords:** Hypofractionation, Prostate cancer, Radiotherapy, Guidelines, Survey

## Abstract

**Purpose:**

Various randomized phase III clinical trials have compared moderately hypofractionated to normofractionated radiotherapy (RT). These modalities showed similar effectiveness without major differences in toxicity. This project was conducted by the Prostate Cancer Expert Panel of the German Society of Radiation Oncology (DEGRO) and the Working Party on Radiation Oncology of the German Cancer Society. We aimed to investigate expert opinions on the use of moderately hypofractionated RT as a definitive treatment for localized prostate cancer in German-speaking countries.

**Methods:**

A 25-item, web-based questionnaire on moderate-hypofractionation RT was prepared by an internal committee. The experts of the DEGRO were asked to complete the questionnaire.

**Results:**

Fourteen active members of DEGRO completed the questionnaire. The questions described indications for selecting patients eligible to receive moderate hypofractionation based on clinical and pathological factors such as age, urinary symptoms, and risk-group. The questions also collected information on the technical aspects of selection criteria, including the definition of a clinical target volume, the use of imaging, protocols for bladder and rectal filling, the choice of a fractionation schedule, and the use of image guidance. Moreover, the questionnaire collected information on post-treatment surveillance after applying moderately hypofractionated RT.

**Conclusion:**

Although opinions varied on the use of moderate-hypofractionation RT, the current survey reflected broad agreement on the notion that moderately hypofractionated RT could be considered a standard treatment for localized prostate cancer in German-speaking countries.

## Introduction

In recent years, hypofractionated radiotherapy (RT), where high doses per fraction are delivered over a relatively short overall treatment duration, has become increasingly popular. Hypofractionated RT has been well established for various tumor entities, including localized prostate cancer. In addition, ultrahypofractionated external beam RT is currently emerging, where doses of 5 Gy or more are delivered per fraction [1, 2]. However, to date, ultrahypofractionation has mainly been performed in clinical trials. Currently, more data are available on moderately hypofractionated RT, which delivers doses between 2.2 and 4 Gy per fraction. Recently, a Cochrane methodology review covered 10 randomized trials, and of those, three provided long-term follow-up data [3]. Fractionation schemes and study endpoints differed among the trials, but the trials showed no differences in oncological outcomes between normal and hypofractionated RT. Moreover, similar rates of disease-specific, metastasis-free, and overall survival were noted, and little or no differences were observed in acute and late toxicity. However, due to the differences in these trials regarding patient characteristics, fractionation, treatment planning, and treatment delivery, an “optimal” protocol for moderately hypofractionated RT remains to be determined.

Therefore, we investigated the current views on moderate hypofractionation among the experts on the Prostate Cancer Expert Panel of the German Society of Radiation Oncology (DEGRO) [1, 4–7], given their expertise and their influence in shaping the direction of future guidelines. Additionally, we have highlighted a few issues such as patient selection and the implementation of moderate hypofractionation for prostate cancer in clinical practice.

## Materials and methods

### Survey design

The Prostate Cancer Expert Panel of DEGRO currently includes 17 active members. We contacted these members to request their participation in a survey on current patterns of practice with moderate-hypofractionation RT in the treatment of patients with prostate cancer. Institutions with more than one member in the expert panel were only allowed to submit one survey to avoid overrepresentation. Thus, 14 experts completed the questionnaire.

The questionnaire was designed by MS and PG and approved by DB and TW. The questionnaire consisted of three parts:

1. Indications for moderately hypofractionated RT
2. Technical aspects (e.g., contouring, planning, and treatment delivery)
3. Follow-up

Prostate risk groups were defined according to the National Comprehensive Cancer Network (NCCN) guidelines, as previously described [6]. Participation in the survey was voluntary, and no financial incentives were offered to participants. Due to the non-interventional nature of the study and the fact that no patients or patient data were included, this survey study did not require ethical approval.

## Results

Fourteen experts (100%) from different centers completed the web-based questionnaire. Twelve experts were working in public academic hospitals and two were working in private facilities. The experts were based in Germany (*n* = 11), Austria (*n* = 2), and Switzerland (*n* = 1; Table 1).

**Table 1:** Survey of the participating experts

Survey population		
Characteristic	Category	*n* (%)
Gender	Male	12 (86%)
	Female	2 (14%)
Country of work	Austria	2 (14%)
	Germany	11 (79%)
	Switzerland	1 (7%)
Institution	Private	2 (14%)
	Public academic	12 (86%)

### Part I: Indications for moderately hypofractionated RT

The expert opinions on the indications for moderately hypofractionated RT are summarized in Table 2.

**Table 2:** Expert responses to indications for moderately hypofractionated radiotherapy

*Is age a criterion for mod. hypo. RT of the prostate?*	
Yes	9 (64%)
No	5 (36%)
*Should there be a minimum age?*	
No minimum age	11 (78%)
70 years	3 (22%)
*Should there be a maximum age?*	
No maximum age	12 (86%)
Yes	2 (14%)
*Is life expectancy a criterion for patient inclusion?*	
No	3 (21%)
Life expectancy >5 years	7 (50%)
Life expectancy >10 years	4 (29%)
*Which risk group is being offered mod. hypo. RT in your institution?*	
Very low risk	7 (50%)
Low risk	12 (86%)
Favorable intermediate risk	13 (93%)
Unfavorable intermediate risk	13 (93%)
High risk	11 (79%)
Very high risk	3 (21%)
*Which prostate volumes are allowed treatment when no lower urinary tract symptoms are present?*	
No criterion	4 (31%)
<60 cc	2 (14%)
<100 cc	8 (62%)
<120 cc	0
*Which prostate volumes are allowed treatment when lower urinary tract symptoms are present?*	
No criterion	5 (38%)
<60 cc	4 (31%)
<100 cc	4 (31%)
<120 cc	0
*Do you offer mod. hypo. RT to patients that underwent TUR-P?*	
No	7 (50%)
Yes	7 (50%)
*What is the minimum time between TUR‑P and mod. hypo. RT?*	
No limit	0
3 to 6 months	2 (14%)
>6 months	5 (50%)
*Should the IPSS of the patient be considered?*	
Yes	14 (100%)
No	0
*What is the maximum IPSS allowed for mod. hypo. RT?*	
<8	1 (7%)
8–10	3 (22%)
10–15	10 (71%)
*Do you offer mod. hypo. RT to patients with pelvic lymph node metastasis?*	
No	11 (78%)
Yes	3 (22%)
*Do you offer mod. hypo. RT to patients with synchronous distant metastasis?*	
No	11 (78%)
Yes	3 (22%)
*Do you offer prophylactic pelvic nodal irradiation with mod. hypo. RT?*	
No	11 (78%)
Yes	3 (22%)
*Is androgen deprivation therapy given in combination with mod. hypo. RT?*	
Yes	13 (93%)
No	1 (7%)

*mod. hypo. RT* moderately hypofractionated radiotherapy, *TUR‑P* transurethral resection of the prostate, *IPSS* International Prostate Symptom Score

Most questionnaire respondents indicated that patients with primary prostate cancer should be treated with moderate-hypofractionation RT, regardless of age group, and that there should be no minimum or maximum age. However, life expectancy seemed to affect the treatment decision. Half of the experts considered moderate hypofractionation appropriate for patients with more than 5 years life expectancy, but only 29% considered it appropriate for patients with over 10 years life expectancy (Table 2).

About 86% of the experts offered moderately hypofractionated RT to low-risk patients, 93% offered it to both favorable and unfavorable intermediate-risk groups, and 79% considered it a treatment option for high-risk patients. Only 21% of experts performed moderate-hypofractionation RT for patients in the very high-risk group.

In the absence of obstructive urinary symptoms, 62% of the questionnaire respondents allowed moderate-hypofractionation RT in patients with prostate volumes ≤100 cc. However, only 31% considered it appropriate when obstructive lower urinary symptoms were present. All experts agreed that the International Prostate Symptom Score (IPSS) of patients should be considered before offering moderate-hypofractionation RT. The majority of experts (71%) offered moderately hypofractionated RT to patients, even when the IPSS sum was 10–15 points. Half of the experts considered a previous transurethral resection of the prostate a contraindication for moderate-hypofractionation RT.

Three centers (22%) currently offer therapeutic pelvic nodal irradiation, in addition to moderate-hypofractionation RT. Additionally, 3 (22%) centers currently offer prophylactic pelvic nodal RT (Table 1).

### Part II: Technical aspects (treatment contouring, planning, and delivery)

All technical aspects of moderate-hypofractionation RT, including contouring, planning, and treatment delivery, are summarized in Table 3. The definition of clinical target volume (CTV) varied among the participating centers, according to the risk category of the patient. For contouring, 11 (78%) experts considered magnetic resonance imaging (MRI) mandatory. Nine (64%) experts apply prostate-specific membrane antigen (PSMA) positron-emission tomography (PET) computed tomography (CT) for staging and/or treatment planning. Thirteen (93%) centers used a predefined protocol for bladder filling and nine (64%) also used a protocol for rectum emptying when planning computer tomography and during treatment. Over half of the experts (64%) preferred the fractionation scheme used in the CHHiP and PROFIT trials (total dose 60 Gy/3.0 Gy per fraction), with its corresponding constraints on doses delivered to target volumes and organs at risk. All centers (100%) considered image-guided RT (IGRT) mandatory for moderately hypofractionated RT. However, only 12 centers (86%) tended to use IGRT on a daily basis (Table 3).

**Table 3:** Expert responses to contouring, planning, and delivery of moderately hypofractionated radiotherapy

*Is MRI mandatory for RT treatment planning?*	
Yes	11 (78%)
No	3 (22%)
*What is the preferred fractionation scheme?*	
60 Gy/20 fractions	9 (64%)
70 Gy/28 fractions	1 (7%)
62 Gy/20 fractions	1 (7%)
Others	3 (22%)
*Is PSMA-PET/CT used?*	
Yes	9 (64%)
No	5 (36%)
*Is IGRT mandatory for the treatment?*	
Yes	14 (100%)
No	0
*What is the frequency of IGRT?*	
Daily	12 (86%)
Others	2 (14%)
*Is fiducial marker implantation mandatory?*	
Yes	6 (43%)
No	8 (57%)
*What is the preferred IGRT technique?*	
Soft tissue matching using CBCT/MVCT without markers	6 (43%)
CBCT/MVCT prostate matching using markers	5 (36%)
Prostate matching using markers without CBCT/MVCT (Electronic portal imaging, x‑rays)	1 (7%)
Bone matching only	2 (14%)
Others	0
*Do you use a specific protocol for bladder filling?*	
Yes	13 (93%)
No	1 (7%)
*Do you use a specific bowel preparation regime?*	
Yes	9 (64%)
No	5 (36%)
*Is rectal spacer Implantation mandatory?*	
Yes	1 (7%)
No	13 (93%)

*MRI* magnetic resonance imaging, *RT* radiotherapy, *PSMA-PET/CT* prostate-specific membrane antigen positron-emission tomography/computed tomography, *IGRT* image-guided RT, *CBCT/MVCT* cone beam computed tomography/megavoltage computed tomography

### Part III: Follow up

The standard follow-up examinations after treatment for primary prostate cancer included evaluations of acute and late genitourinary and gastrointestinal toxicities and quality of life. In addition, 12 centers regularly measured prostate-specific antigen (PSA) at 3 months after the end of moderately hypofractionated RT, and two centers preferred to perform a first PSA assessment at an earlier timepoint.

## Discussion

The current survey shows a broad degree of acceptance for using moderate hypofractionation for treating primary prostate cancer patients among leading radiation oncologists in German-speaking countries. However, significant variations in practicing moderate hypofractionation have been detected.

Ten previous randomized trials have provided strong evidence in support of the non-inferiority of moderate-hypofractionation RT compared to standard normofractionation RT schedules in the treatment of primary prostate cancer [3]. This evidence led to the integration of moderate-hypofractionation schedules into the list of valid treatment options in the NCCN guidelines. However, the European Association of Urology (EAU) [8] and the German S3 guidelines (https://www.leitlinienprogramm-onkologie.de/leitlinien/prostatakarzinom/) only recommend the use of moderate hypofractionation in well-selected patients, with protocols that adhere to the published clinical trials, and in well-equipped centers that offer at least IGRT and intensity-modulated RT (IMRT). The present survey has documented the patterns of practicing moderate-hypofractionation RT in German-speaking countries. This information can provide an additional basis for therapeutic recommendations.

In this survey, 11 out of 14 participants considered it appropriate to offer moderate-hypofractionation RT, independent of age, for patients with a life expectancy greater than 5 years. This result was consistent with the inclusion criteria of published randomized trials. In the PROFIT [9] and CHHiP [10] trials, the median ages were 71 and 69 years, respectively. The median age in the NRG Oncology 0415 trial was 67 years [11]. Moreover, a subgroup of the CHHiP trial included 491 patients aged ≥75 years. They suggested that hypofractionated RT was well tolerated and effective in this subgroup of aged patients [12].

The majority of patients included in the moderate-hypofractionation RT trials had intermediate-risk disease. Those studies provided strong evidence in support of the use of moderate-hypofractionation schedules in that setting. The NRG Oncology 0415 trial presented moderate hypofractionation as a reasonable treatment option for patients with low-risk disease [11] when local treatment was warranted. Less is known regarding the use of moderate-hypofractionation RT for patients with high-risk disease. Notably, the HYPRO trial enrolled around 50% of patients with ≥T3a disease [13]. In the CHHiP trial, only 400 (12%) of the enrolled patients were at a high risk. Patients with very-high-risk disease (Gleason score = 6 with PSA >30, Gleason score = 7 with PSA >20, Gleason score ≥9, and Gleason score ≥8 with T3) were excluded to avoid suboptimal treatment, because androgen deprivation treatment (ADT) was offered for only 6 months [10]. The Arcangeli trial included mostly patients at high risk and showed that 62 Gy, delivered in single 3.1-Gy fractions, was superior to normofractionated RT of 80 Gy (all patients received 9 months of ADT) [14]. The role of nodal irradiation in patients with pelvic lymph node metastasis or elective nodal irradiation for high-risk patients undergoing moderate-hypofractionation RT remains unexplored. However, two questionnaire respondents considered it appropriate to offer pelvic nodal irradiation combined with moderately hypofractionated RT in the prostate, with the simultaneous integrated boost technique.

Previous clinical trials have shown inconsistent results for combining ADT with moderate-hypofractionation RT schedules. The CHHiP trial offered 3–6 months of ADT before and during RT to all patients, independent of their risk group [10]. In contrast, no ADT was given to intermediate-risk patients in the PROFIT trial [9]. The evidence in support of administering a short course of ADT (4–6 months) in patients with unfavorable intermediate-risk disease stemmed from randomized trials that delivered normofractionated RT [6, 12]. For high-risk disease, long-term ADT is well established [8]; however, data are lacking on long-term ADT in combination with hypofractionation RT. Thus, it remains unclear whether prolonging ADT might improve the outcome of moderately hypofractionated RT in high-risk patients.

Both the PROFIT and CHHiP trials delivered 60 Gy in 20 fractions over 4 weeks (in the CHHiP trial, the median duration was 29 days) compared to 74 Gy in 37 fractions. In the present study, most survey participants preferred this hypofractionated schedule, because it was supported by the most robust evidence to date. Although the 60 Gy in 20 fractionation schedule is widely accepted between the experts participating in this survey, two experts are using fractionation schedules published elsewhere, namely 28 × 2.5 Gy (RTOG trial, one expert) and 20 × 3.1 Gy (Arcangeli trial, one expert), and another three experts are using moderate hypofractionation within the frame of internal protocols allowing focal dose intensification in the prostate. Given the higher biological effectiveness of the Arcangeli scheme compared to the CHHiP scheme, the former scheme might be preferred for patients with high-risk disease.

Importantly, intense research is currently exploring the use of focal dose intensification in the prostate. Previous trials have demonstrated that focal dose intensification did not cause excess toxicity [15]. Moreover, after a follow-up of 72 months, one trial found improved tumor control in terms of biochemical disease-free survival [16]. Another, ongoing trial aimed to investigate moderately hypofractionated RT for the prostate alone compared to additional elective RT to the pelvic lymph nodes ± focal dose intensification in the prostate [17]. If these approaches are effective and show favorable oncologic endpoints, the results of these trials might change the standard of care.

In addition, it is crucial to optimize systemic treatments in high-risk patients with good general health and long life expectancies. Various clinical protocols are available for recruiting that type of patient. For example, protocols are available from the ENZARAD study (NCT024 46444), which is testing the combination of conventional ADT and enzalutamide, and the EORTC 1414 study (NCT02799706), which is testing the role of a gonadotropin-releasing hormone antagonist.

We noted that three participating experts in this survey do not consider magnetic resonance imaging (MRI) as mandatory for the clinical routine while practicing moderate hypofractionation. MRI can aid in defining treatment volumes in prostate cancer. In addition, MRI can provide information on extracapsular extension and seminal vesicle involvement [18, 19], and the addition of MRI to CT results in a decrease in interobserver contouring variation and smaller prostate volumes [20]. However, the current ESTRO/ACROP target volume delineation guidelines for prostate cancer also provide recommendations for a CT-only-based contouring approach [20]. It has previously been shown that many CT-based contouring errors can be improved without direct incorporation of MRI data [21].

PSMA PET-CT has recently been well-integrated into staging of recurrent prostate cancer. In the primary setting, the proPSMA trial demonstrated a higher diagnostic accuracy of PSMA PET-CT compared to conventional imaging in high-risk patients with prostate cancer and suggested using PSMA PET-CT for staging in high-risk primary prostate cancer [22]. However, it is not yet clear whether the higher accuracy of PSMA PET-CT has a significant impact on the relevant oncological endpoints. This was clearly reflected in the results of the current survey, where nine centers tend to use PSMA PET-CT for primary staging.

Various guidelines recommend including the base of the seminal vesicles in the treatment volume in cases of intermediate-risk disease [20, 23]. Based on the Roach formula, in the CHHiP trial, the proximal 2 cm of the seminal vesicles was included in the treatment volume for men with >15% risk of invasion [24]. On the other hand, the PROFIT trial used Partin’s table to estimate the risk of seminal vesicle involvement. Accordingly, they included the proximal 1 cm of seminal vesicles in the treatment volume when the risk exceeded 15% [25]. We recommend following current target volume delineation guidelines [20].

When moderate-hypofractionation schemes are applied, the standard of care is IMRT/volumetric modulated arc therapy (VMAT) with daily IGRT. This scheme may include the implantation of fiducial markers and/or tomographic localization with kV or MV portal images or cone beam CT, which is typically used for normofractionated RT [26]. A reduction in the CTV-to-planning target volume (PTV) margin down to 7 mm, or even to 3–5 mm, seems to be acceptable, particularly in combination with daily pretreatment imaging. Reducing the CTV-to-PTV margin is sometimes feasible by continuous intrafractional monitoring and short treatment delivery times, with modulated arcs.

Although the PROFIT and CHHiP trials used the same fractionation schedule, different dose constraints were applied [9, 10]. This difference might have been due to the different contouring methods used. In the PROFIT trial, the rectal and bladder walls (3-mm thickness) were contoured at 18 mm superior and inferior to the CTV; in contrast, in the CHHiP trial, the bladder and rectum were contoured as whole organs (Table 4; [27]). We recommend using the specific dose constraints and delineation details established by clinical trials for the different fractionation schedules.

**Table 4:** Prescription aims in CHHiP and PROFIT trials^a^

	CHHiP^b^	PROFIT
CTV	–	D99 ≥ 60 Gy
PTV	D99 ≥ 57 Gy	D99 ≥ 57 Gy
	D1cc ≤ 63 Gy	D1cc ≤ 63 Gy
	D50 = 60 Gy ± 1%	–
Bladder	V60 ≤ 5%	V46 ≤ 30%
	V48 ≤ 25%	V37 ≤ 50%
	V40 ≤ 50%	–
Rectum^b^	V57 ≤ 15%	V46 ≤ 30%
	V40 ≤ 60%	V37 ≤ 50%
Penile Bulb	V40 ≤ 50%	–
Femoral Head	V40 ≤ 50%	V43 ≤ 5%

^a^In the PROFIT trial, the rectal and bladder wall (3 mm thickness) were contoured 18 mm superior and inferior to CTV while the CHHiP the bladder and rectum were contoured as whole organs

^b^Some constraints were collected via personal communication with CHHiP group. Recommended anorectal dose constraints for hypofractionated radiotherapy by CHHiP trial panel: V20: 85%, V30: 57%, V40: 38%, V50: 22%, V60: 0.01%

Several publications from German-speaking countries have reported the outcome of moderate-hypofractionation RT. A retrospective analysis by Vassis et al. included 55 patients with localized prostate cancer who received moderate-hypofractionation RT (60 Gy in 20 fractions). Those patients were compared to a control group of 55 patients who received normofractionated RT (<78 Gy in 37–39 fractions). Both groups used a simultaneous boost technique. After a median follow-up of 13 months, the groups showed no differences in biochemical progression-free survival, and the toxicity profiles were nearly identical [28]. Schörghofer et al. studied 221 consecutive patients with localized prostate cancer. Those patients were treated with moderate-hypofractionation RT, delivered with different schedules depending on the risk classification. They delivered 60 Gy in 20 fractions for the low-risk group, 63 Gy in 21 fractions for the intermediate-risk group, and 67.5 Gy in 25 fractions for the high-risk group. They demonstrated the feasibility of this risk-adapted approach after a median follow-up of 12 months [29]. Tamihardja et al. described 346 consecutive patients with localized prostate cancer. Those patients were treated with 73.9 Gy or 76.2 Gy in 32 or 33 fractions, respectively, with a simultaneous boost technique. After a median-follow up of 61.8 months, the 5‑year biochemical relapse-free survival was 85.4% for the whole group. Moreover, they observed low rates of late toxicity (cumulative 5‑year late grade 3 genitourinary/gastrointestinal toxicity in 4/1.2% of patients) [30].

Overall, the various randomized trial results have suggested that moderate-hypofractionation RT is non-inferior to conventional RT in terms of efficacy and safety for patients with low- and intermediate-risk prostate cancer. Based on the CHHiP study in the UK, switching to the 20-fraction schedule could result in a reduction in the number of treatment fractions by over 200,000 per year [10]. Moreover, a large population-based study conducted in the US analyzed the total annual cost of external beam RT for localized prostate cancer. Those authors suggested that moderate hypofractionation could potentially save around US$ 160–360 million per year, without impacting survival or the toxicity profile. However, cost analyses in one country may not be readily applicable to other countries [31].

## Conclusion

In accordance with the robust level 1 evidence generated from large phase III trials, the experts who participated in this survey broadly agreed that moderate-hypofractionation RT *can* be considered a standard treatment for localized prostate cancer. However, variations in institutions in German speaking countries were observed in the implementation and application of moderate-hypofractionation RT for the treatment of prostate cancer.
